# Fetal Hemoglobin Modifies the Disease Manifestation of Severe *Plasmodium Falciparum* Malaria in Adult Patients with Sickle Cell Anemia

**DOI:** 10.4084/MJHID.2016.055

**Published:** 2016-11-01

**Authors:** Prasanta Purohit, Siris Patel, Pradeep Kumar Mohanty, Padmalaya Das, Jogeswar Panigrahi

**Affiliations:** 1 Sickle Cell Clinic and Molecular Biology Laboratory, Veer Surendra Sai Institute of Medical Sciences and Research (VIMSAR), Burla, Sambalpur, Odisha, India; 2 Department of Infectious Disease, Asian Institute of Public Health, Bhubaneswar, India; 3 School of Life Sciences, Sambalpur University, Jyotivihar, Burla, Odisha, India

Sickle cell anemia (SCA) and *Plasmodium falciparum* malaria are two major public health problems in the state of Odisha, India. The prevalence of sickle cell gene in the western part of Odisha is 13.1%,^1^*P. falciparum* contributes 23% of cases and 15% of malaria-related deaths in India. Various African studies have shown that, even though SCA protects from *P. falciparum* infection, the risk of severe illness and death due to malaria is higher.^2^,^3^ Though several factors are responsible for the disease severity in *P.falciparum* malaria in patients with SCA, it was recently found that fetal hemoglobin (HbF), a physiological hemoglobin usually found higher in patients with SCA had a negative epistatic interaction with HbS during protection against malaria.^4^ The role of HbF against *P. falciparum* malaria in cases with normal hemoglobin genotypes has been widely studied and found to be protective against severe disease manifestation. So it is necessary to investigate this association in the regions with high prevalence of sickle cell gene and high endemicity of *P. falciparum* malaria. This study aims to find out the effect of HbF level on the clinical manifestation of severe *P.falciparum* malaria in patients with SCA.

This prospective study was undertaken at the Sickle Cell Clinic and Molecular Biology Laboratory, Veer Surendra Sai Institute of Medical Sciences and Research, Burla, Odisha, India. Forty-six adult patients with SCA along with severe *P. falciparum* malaria admitted in the Department of Medicine of this institute were included in this study. The mean age of patients was 25.4±8.8 years (range, 17 to 60 years) with 58.7% (27/46) being males. The hemoglobin variants including HbF was estimated by Cation-Exchange high performance liquid chromatography (CE-HPLC) using Variant II −*β*-thalassemia short program (Bio-Rad laboratories, Hercules). The mean % HbF level was found to be 18. 2 ± 4.9 %; ranging from 6.0 to 29.0 %.

The severity of malaria was defined by WHO guideline in 2010.^5^ The severity due to the malarial infection was defined by the presence of single or multiple complications. Cerebral malaria, severe malarial anemia, jaundice, acute renal failure and/or hepatopathy were considered as the major clinical symptoms of the patients. Among the various clinical symptoms, the incidence of cerebral malaria was 37.0% (17/46) followed by severe malarial anemia (21.7%, 10/46). Episodes of vaso-occlusive crises were observed in 50.0% of cases. Death was recorded in 9 patients including six females. The demographic and clinical features of patients have been shown in Table 1. There were multiple complications responsible for mortality in these patients. The comparison of % HbF level in patients with a various number of complications they had revealed that the mean % HbF levels increased with the number of clinical complications in the patients. The average increase in the % HbF level was 15.7±4.0, 18.0±4.0, 18.9±6.1 and 20.8±1.2 respectively in patients with single, two, three and four complications. Further linear regression analysis between total hemoglobin level and % HbF level in the patients elucidated an inverse relationship (*r*, −0.356; *p*, 0.015), which indicates that patients with higher % HbF level had lower total hemoglobin level.

In another comparison, we found that there was a trend in increasing % HbF level in patients with severe malarial anemia compared to patients without it. A similar tendency was observed in patients with cerebral malaria. The average % HbF level also significantly increased averagely in patients who died compared to patients who survived (*p*, 0.01). The % HbF differences in the patients with severe malarial anemia, cerebral malaria, and fatality, has been illustrated in Figure 1.

Like HbS, alpha thalassemia has also been found to have a protective role against *P. falciparum* malaria. However, this protection afforded by alpha thalassemia becomes relatively sluggish when co-inherited with HbS. Because of a higher prevalence of alpha thalassemia in the study area,^1^ we have attempted to compare the hematological and clinical parameters in patients with heterozygous and homozygous alpha thalassemia separately against patients with normal alpha globin genotype. There were no statistically significant differences between the groups in hematological (Supplementary Table 1) as well as clinical parameters except for severe malarial anemia which was higher in patients with homozygous alpha thalassemia (Supplementary Table 2). Severe malarial anemia was associated with alpha thalassemia whereas alpha thalassemia has no impact on mortality, so we have analyzed the association of severe malarial anemia with mortality and was found insignificant (OR, 0.2; 95%CI [0.038–0.98]; *p*, 0.06).

From the three factors above, which are associated with severity and mortality due to *P. falciparum* malaria in patients with SCA, derived that HbF has a negative role in protection against severe disease manifestation. It has been found that HbF provides protection from *P. falciparum* malaria by diminishing the growth of the parasites inside the RBCs.^6^ Instead of giving protection, the high level of HbF in our patients is associated with a major severity of *P. falciparum* infection. This might be due to the peripheral selection and not for its increased synthesis of HbF in patients with SCA.

Though, HbF in SCA is found to be supportive in reducing episodes of vaso-occlusive crises and requirements of blood transfusion,^7^ the protection afforded by HbS against severe malaria reduced with increased % HbF level. This study agrees with the hypothesis of a negative epistatic interaction between HbS and HbF in reducing protection against severe malaria by Mmbando et al.,^4^ In this situation, use of hydroxyurea (a drug which usually increases HbF level) in patients with SCA in malaria endemic regions is debatable. In general, hydroxyurea must be started in severe patients with SCA because (1) increasing HbF level following hydroxyurea therapy is the principal but not the sole determinant of clinical responses in these patients; (2) some patients with minimal or no increased in HbF level following hydroxyurea therapy also showed significant clinical responses.^8^ In the present study, 26 patients were on hydroxyurea therapy, from which 15.4% (4/26) of patients died compared to 25.0% (5/20) of death in patients without hydroxyurea therapy. A large cohort study in a malaria endemic region is essential to give conclusive results on the association of HbF and use of hydroxyurea in patients with SCA.

## Supplementary materials

**Supplementary Table 1:** Comparison of hematological parameters on the basis of alpha globin genotype in severe *Plasmodium falciparum* patients with sickle cell anemia.

Parameters	Normal alpha globin genotype (αα/αα) N=17	Heterozygous Alpha thalassemia (−α/αα) N=20	Homozygous Alpha thalassemia (−α/−α) N=9	*p value*
**Complete blood counts**				
Hemoglobin (g/dL)	7.7±1.8	7.1±2.6	7.1±2.6	0.741
WBC (103 /μl)	8.1±3.8	10.2±7.8	8.4±5.7	0.133
RBC (106 /μl)	2.9±0.5	3.0±1.2	3.0±0.6	0.717
MCV (fL)	87.3±12.0	78.6±13.0	71.0±7.8	0.009
MCH (pg)	28.9±3.9	25.7±3.8	25.0±3.7	0.017
MCHC (g/dL)	32.0±2.3	31.8±2.2	31.3±1.9	0.631
Platelets (103 /μl)	197.2±91.1	192.9±157.8	179.7±70.2	0.554
**Biochemical parameters**				
Glucose (U/L)	106.0±32.6	105.4±28.7	87.9±45.2	0.657
Urea (mg/dL)	34.2±22.3	36.3±25.6	46.2±41.7	0.094
Creatinine (mg/dL)	1.41±0.99	0.94±0.8	1.5±0.9	0.064
SGOT (U/L)	74.2±40.5	71.6±56.0	54.0±28.8	0.295
SGPT (U/L)	54.3±40.0	43.8±34.0	40.6±12.7	0.771
Bil-T (mg/dL)	3.1±1.7	4.2±2.7	3.8±1.8	0.301
Bil-D (mg/dL)	1.1±0.6	1.0±0.9	1.3±0.6	0.253
**Hb Variants by HPLC**				
HbA2 (%)	2.77±0.4	2.7±0.8	2.9±0.5	0.796
HbF (%)	19.7±3.9	18.3±5.2	17.4.1±3.7	0.635
HbS (%)	75.0±3.6	76.2±5.3	75.9±1.5	0.765

WBC, white blood corpuscle; RBC, Red blood corpuscle; MCV, mean corpuscular volume; MCH, mean corpuscular Hb; MCHC, mean corpuscular Hb concentration; HCT, hematocrit; SGOT, Aspartate transaminase; SGPT, alanine transaminase; Bil-T, bilirubin total; Bil-D, bilirubin direct; LDH, lactate dehydrogenase. Comparison between the groups were made by one way analysis of variance by using SPSS version 16.0 for window as statistical software.

**Supplementary Table 2:** Comparison of clinical features on the basis of alpha globin genotype in severe *Plasmodium falciparum* patients with sickle cell anemia.

Clinical signs and symptoms	Total (N=46)	αα/αα (N=17)	−α/αα (N=20)	Odd Ratio 95% CI	−α/−α (N=9)	Odd Ratio 95% CI
Severe Malarial Anemia	10 (21.7)	1 (5.9)	5 (25.0)	0.19 (0.02–1.8)	4 (44.4)	0.08 * (0.007–0.87)
Cerebral Malaria	17 (37.0)	7 (41.2)	8 (40.0)	1.05 (0.28–3.93)	2 (22.2)	2.45 (0.38–15.5)
Hepatopathy	10 (21.7)	4 (23.5)	6 (30.0)	0.72 (0.16–3.13)	0	6.33 (0.30–132.2)
Acute Renal Failure	5 (10.9)	3 (17.6)	1 (5.0)	4.07 (0.38–43.4)	1 (11.1)	1.71 (0.15–19.4)
Jaundice	20 (43.5)	6 (35.3)	9 (45.0)	0.67 (1.18–2.52)	5 (55.6)	0.43 (0.08–2.27)
Fatal Outcome	9 (19.6)	4 (23.5)	3 (15.0)	1.74 (0.33–9.19)	2 (22.2)	1.07 (0.16–7.42)

The comparison has been analyzed separately for heterozygous (−α/αα) and homozygous (−α/−α) alpha thalassemia against normal alpha genotype. The data were presented as number (percentage).

* *p* < 0.05

## Figures and Tables

**Figure 1:**
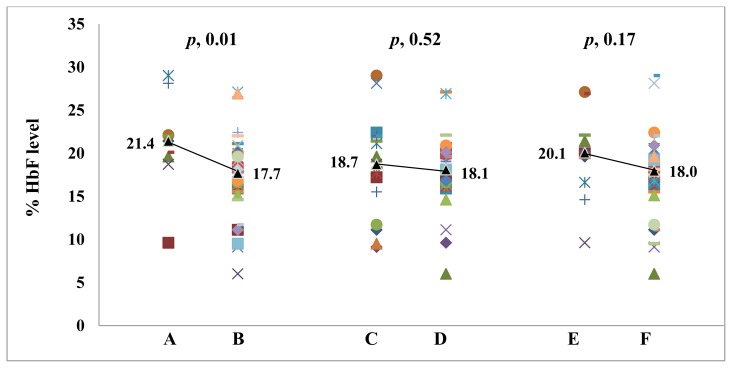
Comparison of % HbF level in patients with severe *Plasmodium falciparum* malaria with different clinical manifestation. **A.** HbF level in death patients; **B.** HbF level in survived patients; **C.** HbF level in patients with cerebral malaria; **D.** HbF level in patients with no cerebral malaria; **E.** HbF level in patients with severe malarial anemia; **F.** HbF level in patients with no severe malarial anemia. The line joined the median value in each category.

**Table 1:** The demographic and clinical features of severe *Plasmodium falciparum* malaria parients with sickle cell anemia (N=46).

Characteristics	Severe *P. falciparum* malaria patients with sickle cell anemia (N=46) Number (%)
Age in year	25.4±8.8 (Range, 17–60 years)
Gender	
Male	27 (58.7)
Female	19 (41.3)
Hemoglobin (mg/dL)	7.4±2.3
HbF (%)	18.2±4.9
Severe Malaria Anemia	10 (21.7)
Cerebral Malaria	17 (37.0)
Hepatopathy	10 (21.7)
Acute Renal Failure	5 (10.9)
Jaundice	20 (43.5)
Episodes of Vaso-Occlusive Crises	23 (50.0)
Fatal Outcome	9 (19.6)

Note: Age, Hemoglobin (mg/dL) and HbF (%) were represented as mean ±SD.
